# Cortical auditory potentials and cognitive potentials in individuals with and without vestibular dysfunction

**DOI:** 10.12688/f1000research.122677.3

**Published:** 2023-10-18

**Authors:** Kaushlendra Kumar, Krishnapriya S, Anupriya Ebenezer, Mohan Kumar Kalaiah, Deviprasad D

**Affiliations:** 1 Department of Audiology and Speech Language Pathology, Kasturba Medical College, Mangalore, Manipal Academy of Higher Education, Manipal, India; 2 Department of Otorhinolaryngology, Kasturba Medical College, Mangalore, Manipal Academy of Higher Education, Manipal, India

**Keywords:** cognition, vestibular dysfunction, vertigo, P300, dizziness, event related potentials, cortical auditory evoked potentials, VEMP

## Abstract

**Background:** Among individuals with vestibular dysfunction, the loss of vestibular sensory information is found to alter cognitive abilities that coordinate spatial and non-spatial information. P300 is an event-related potential commonly used to assess cognitive processing. The aim of the present study was to compare the latency and amplitude of cortical auditory evoked potential and P300 between individuals with vestibular dysfunction and individuals with no vestibular dysfunction.

**Methods:** Forty adults with a mean age of 40.5 ± 13.07 participated in the study. Group I included 20 adults diagnosed with vestibular dysfunction and group II included 20 age-matched adults with no vestibular dysfunction. The P300 was recorded from the electrode site Cz and Pz. It was elicited using pure-tones in odd-ball paradigm. The latency and amplitude of peaks P1, N1, P2, and N2 of the cortical auditory evoked potential and the P300 were measured.

**Results:** Significant amplitude difference was observed in cortical potentials at Cz and Pz. The P300 was present only in 70% of individuals with vestibular dysfunction compared to 100% among individuals with no vestibular dysfunction. The mean amplitude of the P300 was slightly larger in group 1 compared to group 2 and the mean latency of the P300 was similar in both groups. However, the difference in amplitude of the P300 between groups was not statistically significant.

**Conclusions:** Knowing the cognitive function of individuals with vestibular dysfunction enables planning vestibular rehabilitation therapy, which enhances the quality of life in these individuals by improving their vestibular and cognitive functions.

## Introduction

Vestibular dysfunction is caused by pathologies in the peripheral and central vestibular system. The peripheral pathologies constitute 90% of cases with vertigo. ^1^ It involves lesion in the end organs of the inner ear and/or the eighth cranial nerve. The central vestibular pathologies involve lesion in the cortical and sub-cortical pathways of the vestibular system. Vestibular dysfunction results in several adverse physical outcomes such as postural instability, abnormal gait and falls. Further, the majority of individuals with vestibular dysfunction are also found to have anxiety and depression. ^2^ In addition, the loss of vestibular sensory information is shown to alter cognitive abilities related to the processing of spatial and non-spatial information. ^3^

Several studies have investigated the cognitive abilities of individuals with vestibular dysfunction. According to literature, parabrachial nucleus and the hippocampus are the anatomically two regions that account for the relation between the vestibular system and neural networks involved in cognitive and emotional processing. ^4^ The different cognitive skills associated with vestibular function include attention, visuospatial orientation, executive function, memory, metacognition, and self-control. ^5^ Research on cognition assessment pertaining to vestibular function has mainly been based on spatial orientation, attention, memory, and executive function. ^5^ Smith (2017) ^6^ reported that cognitive impairment is usually seen in any vestibular dysfunction such as either peripheral or central vestibular dysfunction.

The P300 is an event-related potential, elicited when the target stimuli in the odd-ball paradigm is identified by the participant. It serves as an index for the assessment of cognitive ability to assess cerebral information processing in the context of various neurological diseases. ^7^ Several studies have documented abnormal P300 in individuals with cognitive dysfunctions such as autism spectrum disorder, ^8^ attention deficit hyperactivity disorder, ^9^ schizophrenia, ^10^ and migraine. ^11^ It is usually performed with minimum attention to the stimuli and without secondary tasks. The P300 is used to evaluate age-related cognitive dysfunction, reflecting attention and memory processes and overlapping function in cognitive deficit. ^12^ Different areas of the brain that generate P300 response include subcortical structures, auditory cortical regions, frontal lobe and various association areas. ^13^ The subcomponents of P300, P3a and P3b are generated by different neural structures. The frontal lobe is the primary generator of P3a, reflecting the working memory, which helps in early attention. The P3b reflects an attention-driven stimulus-generated response and is generated from the temporal and parietal structures. ^7^ The P300 amplitude response mainly depends on stimulus probability, stimulus significance, task effort, motivation, and attentiveness. ^14^ P300 amplitude is directly related to the amount of attention paid to perform a particular task associated with superior memory performance. ^15^ P300 latency reflects stimulus processing time in contrast to response processing time, which corresponds to stimulus evaluation time and is independent of the response section. ^16^^,^^17^

Behavioural cognitive abilities are reported to be affected in individuals with vertigo. ^5^^,^^18^ Especially in individuals with vestibular dysfunction, the cognitive deficit is not only observed in terms of spatial orientation, but also with respect non spatial activities which includes recognition memory and attention. Whereas, the authors had suggested that there might be an inverse proportionality between the cognitive dysfunction and frequency of vertigo. Therefore, the individuals who achieved vestibular compensation might also experience cognitive deficit. ^19^ Moreover, considering the findings of the previous research there is a necessity to study the direct measure of brain response to cognitive task by recording an event related potentials. Especially the cortical potentials to read the sensory aspects and cognitive potentials to read the cognitive aspects of individuals with vestibular dysfunction. Hence, the current study aims to compare the findings of cortical potentials (P1, N1, P2, and N2) and cognitive potentials (P300 and N4) between individuals with and without vestibular dysfunction. The objective was to investigate the relationship of cortical and cognitive potentials peak latency and peak amplitude in individuals with and without vestibular dysfunction and the correlation between DHI score with P300 findings among individuals with vestibular dysfunction.

## Methods

### Participants

A total of 40 adults aged between 20 and 60 years (mean = 40.5, SD = 13.1) participated in this study. Group I included 20 adults, (mean = 40.5 years, SD = 13.1) 10 males and 10 females with vestibular dysfunction. All participants in group I had underwent detailed vestibular evaluation. The tests administered were subjective vestibular assessment, oculomotor examination, and vestibular evoked myogenic potentials (VEMPs). The oculomotor examination was performed using videonystagmography (VNG), the subtests included were saccade test, tracking test, and optokinetic test. VEMP testing included both cervical VEMP (cVEMP) and ocular VEMP (oVEMP). The patients having abnormal findings on oculomotor examination or VEMPs assessment was considered for this study. Group II included 20 age-matched adults (mean = 40.5 years, SD = 13.1) 10 males and 10 females with no vestibular dysfunction. Twenty individuals with vestibular dysfunction in group I included 12 individuals diagnosed with peripheral vestibular lesion and eight individuals with central vestibular lesion. All participants in the study had hearing sensitivity within normal limits in both ears with pure-tone average (500 Hz, 1000 Hz, 2000 Hz, and 4000 Hz) less than 25 dBHL. The mean pure-tone average was 18.18 dBHL (SD = 5.16) for right ear, 18.20 dBHL (SD = 5.58) for left ear in group I and was 15.96 dBHL (SD = 4.59) for right ear, 16.43 dBHL (SD = 3.69) for left ear in group II. Individuals under medication for vertigo and individuals diagnosed as having an autoimmune disease, systemic illness or neurodegenerative disorders were excluded from the study. The study was approved by the institutional ethics committee of Kasturba Medical College, Mangalore (IECKMCMLR11-18/456) and written informed consent was obtained from all participants before they participated in the study.

### Dizziness Handicap Inventory (DHI)

All participants in group I completed a Dizziness Handicap Inventory (DHI) questionnaire. ^20^ It assesses quality of life of participants on three domains: functional (nine questions), emotional (nine questions) and physical (seven questions). The participants were instructed to provide responses such as “Yes” when the symptom is present always, “Sometimes” when the symptoms is present sometimes, and “No” when it is absent. Item scores were summed, and the maximum score was 100 and the minimum score was 0. Answers were graded according to 0 for a “No” response, 2 for a “Sometimes” response and 4 for a “Yes” response.

### Recording of P300

The P300 was recorded using the IHS Smart EP version 3.92 evoked potential system (Intelligent Hearing Systems, USA). During the recording of the P300, participants were made to sit comfortably on a reclining chair in a sound-treated room. The electrode sites were cleaned using Nu-prep Skin Prep Gel (Weaver and Company, USA). Gold plated disc electrodes were placed on the electrode sites using conduction paste and it was secured using adhesive tape. Two non-inverting electrodes were placed on the scalp, one on the vertex (Cz) and the other on the parietal (Pz). Inverting electrodes were placed on both ear mastoid (linked mastoid), and the ground electrode was placed on low forehead (Fpz). The electrode impedance was maintained below 5 kΩ for each electrode and the inter-electrode impedance was less than 2 kΩ. The P300 was elicited using pure-tones of 1000 Hz and 2000 Hz in an odd-ball paradigm. The 1000 Hz pure-tone served as standard stimuli (80%) and the 2000 Hz pure-tone served as deviant stimuli (20%). The standard and target stimuli were presented at a ratio of 4:1. The pure-tones were presented to both ears of participants at 80 dB SPL using ER-3A insert earphones (Intelligent Hearing Systems, USA). A total of 300 stimuli were presented at a repetition rate of 1.1 stimuli/sec and the ongoing EEG was differentially recorded from the scalp. The EEG was amplified 50,000 times and filtered using a bandpass filter of 1 to 30 Hz. Sweeps with amplitude greater than ±50 µV were rejected from averaging. The duration of the analysis window was 600 msec with a pre-stimulus duration of 100 msec. The participants were instructed to count the target stimuli and report at the end of the recording. All participants had a practice trial on the task before ERPs were recorded.

### Data analysis

The waveforms obtained from all participants for standard and target stimuli were grand averaged separately to identify various components or peaks of event related potentials. The averaged waveforms included peaks P1, N1, P2, and N2 between 50 msec and 250 msec. The broad positive peak after 250 msec from the stimulus onset in the waveform of target stimuli was referred to as P300. And the negativity peak following the P300 was considered as N4 (late negativity). The latency (in msec) and peak amplitude (in μV) of peaks P1, N1, P2, N2, P300 and N4 was measured at the electrode sites Cz and Pz. The peak amplitude was measured relative to the pre-stimulus baseline and the latency was measured from the stimulus onset. The dataset is published as underlying data in Mendeley Data. ^21^

### Statistical analysis

Statistical analysis was conducted using IBM SPSS software version 26 (RRID:SCR_002865) *.* Initially, descriptive analysis and the Shapiro-Wilk test were carried out to check the normality of the data. The latency and peak amplitude of peaks P1, N1, P2, N2, P300, and N4 were normally distributed. Thus, the independent t-test was administered to investigate if the mean latency and amplitude of peaks were significantly different between groups. The P300 association between group I and II was analysed using chi-square test. Pearson’s correlation analysis was carried out to investigate the relationship between the DHI score and latency and amplitude of P300 in group I.

## Results

**Figure 1.**
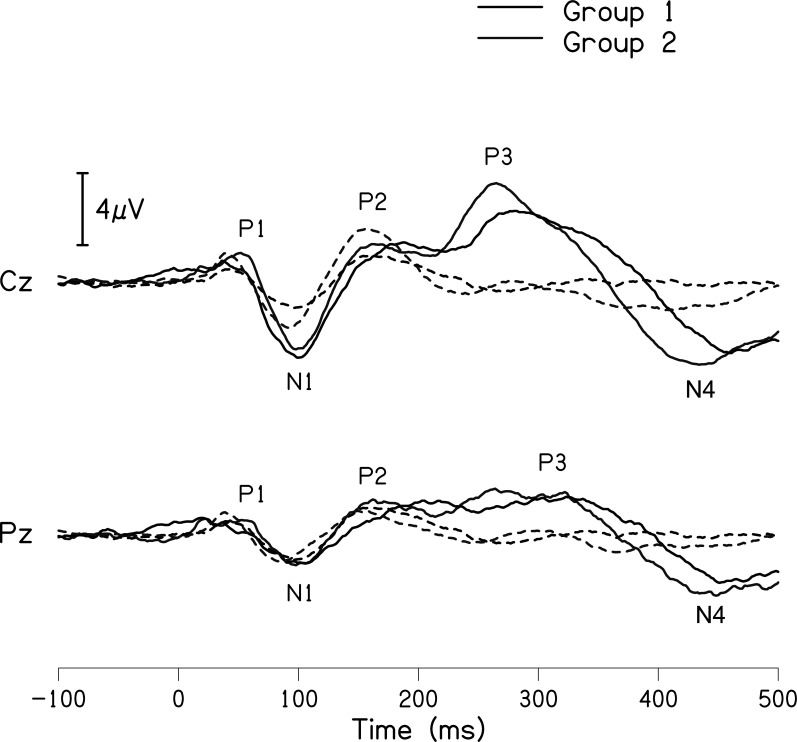
Grand average waveforms of P300 recorded at Cz and Pz for 1000 Hz standard tone (dashed line) and 2000 Hz deviant tone (solid line) for group I (black) and group II (gray).

**Figure 2.**
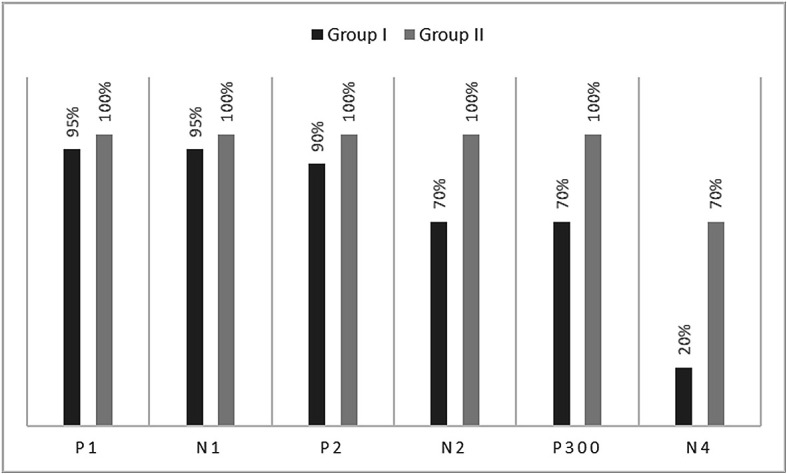
Identification rate of peaks P1, N1, P2, N2, P300 and N4 at Cz position in both the groups.

**Figure 3.**
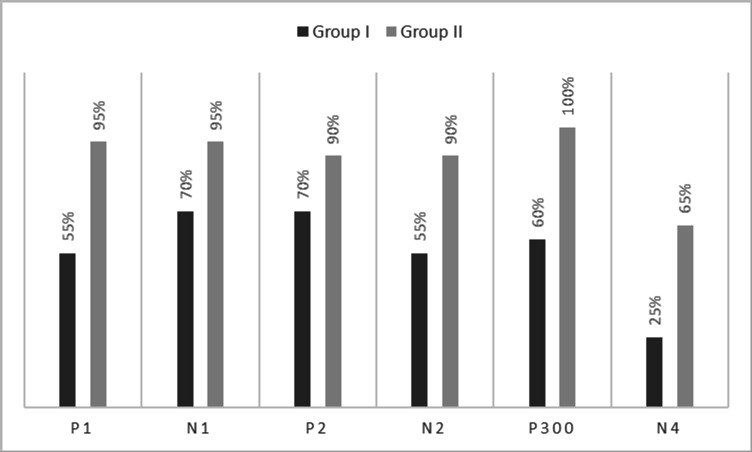
Identification rate of peaks P1, N1, P2, N2, P300 and N4 at Pz position in both the groups.

The grand averaged waveforms for standard and deviant stimuli for both groups are shown in Figure 1. In the figure, it is evident that both pure-tones elicited obligatory P1-N1-P2 response for the stimulus onset among participants in both groups. Further, the waveform for target stimuli showed a large positive peak at a latency of 280 ms following the P1-N1-P2 response in both groups of participants, referred to as P300. The identification rate of peak responses varied across groups at Cz and Pz positions, which is depicted in Figures 2 and 3 respectively. The P300 was absent in a greater number of individuals with vestibular dysfunction compared to the control group. To investigate the association between vestibular dysfunction and P300, a chi-square test was carried out. The result showed a statistically significant association between vestibular dysfunction and the presence or absence of P300 (χ ^2^(1) = 8.53, p = 0.003) with an odd ratio of 15.83.

Table 1 shows the mean latency of peaks P1, N1, P2, and N2 at electrode sites Cz and Pz for both groups. The mean latency of peaks P1, N1, P2, and N2 were similar between groups at the electrode sites Cz and Pz. To investigate if the mean latency of peaks were significantly different between groups, the independent t-test was carried out. It showed no significant difference for the latency of peaks P1 [t(37) = -1.083, p = 0.286)], N1 [t(37) = -1.008, p = 0.320)], P2 [t(36) = 1.007, p = 0.321)], and N2 [t(32) = 1.822, p = 0.078)] at the electrode site Cz. Similarly, at Pz the latency of peaks P1 [t(28) = -0.029, p = 0.977)], N1 [t(31) = -0.029, p = 0.977)], P2 [t(30) = -1.059, p = 0.298)], and N2 [t(27) = 0.56, p = 0.956)] were not significantly different between groups.

**Table 1.** Descriptive analysis of peaks P1, N1, P2 and N2 latency and amplitude at Cz position.

	Latency (msec)				Amplitude (μV)			
	*P1*	*N1*	*P2*	*N2*	*P1*	*N1*	*P2*	*N2*
**Group I (Cz)**								
Mean	55.50	105.63	168.20	226.07	0.86	3.69	2.44	1.22
SD	5.16	7.52	15.58	16.71	1.58	2.81	1.95	1.97
n	19	19	18	14	19	19	18	14
**Group II (Cz)**								
Mean	57.70	107.70	163.35	214.10	2.34	-3.15	3.09	1.35
SD	7.15	5.12	14.57	20.18	1.78	2.69	1.85	0.85
n	20	20	20	20	20	20	20	20

Table 2 shows the mean amplitude of peaks P1, N1, P2, and N2 at electrode sites Cz and Pz for both groups. The mean amplitude of peaks was larger in individuals with no vestibular dysfunction (group II) at both Cz and Pz. To investigate if the mean amplitudes of peaks are significantly different between groups, independent t-test was carried out separately for Cz and Pz. It revealed no significant difference for the amplitude of peaks P1 [t(30) = -2.733, p = 0.10)], N1 [t(37) = -0.614, p = 0.543)], P2 [t(36) = -1.061, p = 0.296)] and N2 [t(32) = -0.268, p = 0.790)] at the electrode site Cz between groups. Similarly, the amplitude of peaks P1 [t(28) = -1.742, p = 0.093)], N1 [t(31) = -1.326, p = 0.194)], P2 [t(28) = -1.742, p = 0.093)] and N2 [t(27) = -0.318, p = 0.756)] at the electrode site Pz showed no significant difference between the groups. On peak-to-peak analysis between groups showed no significant difference in P1-N1 and N1-P2 peak to peak amplitude at Cz and Pz.

**Table 2.** Descriptive analysis of peaks P1, N1, P2 and N2 latency and amplitude at Pz position.

	Latency (msec)				Amplitude (μV)			
	*P1*	*N1*	*P2*	*N2*	*P1*	*N1*	*P2*	*N2*
**Group I (Pz)**								
Mean	62.81	110.00	163.92	227.36	1.36	-2.38	1.69	1.06
SD	10.64	8.63	16.87	23.20	0.79	2.17	0.88	1.26
n	11	14	14	11	11	14	14	11
**Group II (Pz)**								
Mean	62.94	110.10	170.50	226.94	2.17	-1.44	2.79	1.22
SD	12.60	11.60	17.82	17.32	1.40	1.87	1.28	1.32
n	19	19	18	18	19	19	18	18

Table 3 shows the mean latency and amplitude of peaks P300 and N4 at Cz and Pz for both groups. A noticeable difference was observed for the mean amplitude of N4 between groups at both the electrode sites. The mean amplitude of P300 was larger in group I compared to group II, this finding is contrary while comparing the grand average waveform of both groups shown in Figure 1. The mean amplitude of P300 was found to be largest in group 1 compared to group II. To investigate if the mean difference for latency and amplitude were significantly different between groups, an independent samples t-test was carried out. The results showed no significant difference in the latency of P300 [Cz: t(32) = -0.173, p = 0.866); Pz: t(30) = -0.357, p = 0.724)] and amplitude of P300 [Cz: t(32) = 0.218, p = 0.829); Pz: t(29) = 0.797, p = 0.432)] at both Cz and Pz. Whereas no statistical significant difference was found for the N4 latency [Cz: t(16) = 0.415, p = 0.684); Pz: t(16) = 0.786, p = 0.443)] and significant difference was observed in N4 amplitude [Cz: t(14) = -2.178, p = 0.047); Pz: t(15) = -2.107, p = 0.052)] at both positions.

**Table 3.** Descriptive analysis of peaks P300 and N4 latency and amplitude at Cz and Pz positions.

	Latency (msec)				Amplitude (μV)			
	Cz		Pz		Cz		Pz	
	*P300*	*N4*	*P300*	*N4*	*P300*	*N4*	*P300*	*N4*
**Group I**								
Mean	344.07	440.75	344.83	449.00	4.46	-0.01	3.72	0.87
SD	26.02	6.84	21.70	21.37	4.97	6.32	3.17	4.49
n	14	4	12	5	14	4	12	5
**Group II**								
Mean	345.55	436.14	347.85	439.23	4.16	-6.66	3.06	-4.86
SD	23.39	21.47	23.93	24.30	2.98	4.99	1.41	3.13
n	20	14	20	13	20	14	20	13

To investigate the relationship between the latency of P300 and DHI score, Pearson’s correlation analysis was carried out. The results showed a weak negative correlation between the latency of P300 and the DHI score; however, the correlation was not significant at both electrode sites [Cz: r = -0.201, p = 0.490; Pz: r = -0.401, p = 0.196]. Further, no correlation was found between the amplitude of the P300 and DHI score at both electrode sites [Cz: r = -0.167, p = 0.569; Pz: r = 0.087, p = 0.787].

The mean latency of late latency response and P300 of individuals diagnosed with peripheral vestibular lesion and central vestibular lesion are depicted in Tables 4 and 5 respectively.

**Table 4.** Descriptive analysis of peaks P1, N1, P2, N2 and P300 latency among individuals with peripheral vestibular lesion.

Latency	Cz			Pz		
*msec*	*n*	*Mean*	*SD*	*n*	*Mean*	*SD*
**P1**	11	56.09	5.12	6	65.33	12.50
**N1**	11	104.54	9.63	7	107.71	9.12
**P2**	10	166.00	14.03	7	167.42	16.71
**N2**	8	230.37	15.74	5	235.20	13.19
**P300**	9	345.22	25.49	7	349.57	25.99

**Table 5.** Descriptive analysis of peaks P1, N1, P2, N2 and P300 latency among individuals with central vestibular lesion.

Latency	Cz			Pz		
*msec*	*n*	*Mean*	*SD*	*n*	*Mean*	*SD*
**P1**	8	54.75	5.47	5	59.80	8.19
**N1**	8	107.12	2.90	7	112.28	8.13
**P2**	8	171.12	17.88	7	160.42	17.58
**N2**	7	188.85	84.81	6	220.83	28.72
**P300**	5	342.00	29.88	5	338.20	13.68

## Discussion

The present study compared the latency and amplitude of cortical (P1, N1, P2 and N2) and cognitive potentials (P300) among individuals with and without vestibular dysfunction. The cortical potential includes auditory late latency responses P1, N1, P2, and N2. As per a review, it is clear that cognitive impairment is observed in individuals with vestibular dysfunction, with respect to attention, spatial orientation, executive function and memory. ^5^

The results of the present study showed no significant difference in the latency and amplitude of peaks P1, N1, P2, and N2 of the cortical auditory potentials. The cortical findings of the present study could be explained based on the hearing sensitivity of participants in both groups and the characteristics of the P1-N1-P2 response. The P1-N1-P2 response is elicited for the onset of stimuli, therefore, the characteristics of the response are dependent on the onset of the stimuli. Further, the participants in both groups had hearing sensitivity within normal limits. Thus, the latency and amplitude of the peaks are expected to be similar in both groups.

The P300 was found to be absent in a greater number of individuals with vestibular dysfunction compared to the control group. It was absent in 30–40% of the individuals with vestibular dysfunction; this finding is consistent with the results of the previous study. ^22^ Further, when the P300 was present, the mean latency and amplitude of the P300 in both groups were similar. In contrast to the findings of the present study, earlier investigations have reported prolonged latency for P300 in individuals with vestibular dysfunction compared to the control group. ^23^ The contrasting findings observed in the present study and earlier investigations could be because of differences in the site of vestibular lesion across studies. In the literature studies majorly included individuals with abnormal caloric function suggestive of having either unilateral or bilateral lateral semicircular canal dysfunction. ^23^^,^^24^ The findings of the earlier investigation showed prolongation of P300 latency in individuals with unilateral caloric hypofunction compared to the control group. ^24^ Whereas, the participants in the present study had peripheral vestibular lesion with abnormal findings on cVEMP and oVEMP indicating a lesion in the saccule and utricle. Therefore, the contrasting findings observed the difference in the P300 findings of the present study could be a consequence due to variation in the site of the lesion. In addition, contrasting findings observed in the present study could be also due to the degree of dizziness handicap due to vestibular dysfunction. The degree of handicap might have an influence on the latency and amplitude of P300 showing a direct proportionality that higher the degree of dizziness, higher is the cognitive impairment. ^25^

Studies on cognitive function assessment using a cognitive failure questionnaire in individuals with vestibular dysfunction revealed that cognitive dysfunction is prevalent in individuals with central and peripheral vestibular pathologies. ^26^ The literature also showed a positive correlation between cognitive dysfunction and dizziness handicap in terms of a self-rated questionnaire. DHI helps in evaluating the dizziness handicap based on its impact physically, functionally, and emotionally with a limited profile on cognition. ^27^ In the current study, the correlation between DHI scores and P300 showed no significant correlation. The lack of correlation between the two measures could be due to the different areas of assessment. DHI is a measure of self-help obtained primarily based on daily activities, whereas the P300 is an electrophysiological measure that assesses cognitive functioning. ^13^ Because of this direct correlation between DHI and P300 was found to be inconclusive in the present study. Similarly, no significant correlation was observed between P300 and vertigo symptoms, whereas another study stated that severity of vestibular symptoms seems to correlate with P300 responses. ^28^ In support of the current study, a randomized controlled trial showed cognitive behaviour therapy influenced patients with chronic subjective dizziness with a significant reduction in DHI and no changes in psychological outcome measures. ^29^ Similarly, other literature has reported that the functional and physical parameters of DHI showed a negative correlation, and the emotional parameter showed a weak significant positive correlation in 369 participants evaluated for functional tests such as electronystagmography, rotational testing, and platform posturography. ^30^ The findings of various studies by several investigators have emphasized the role of the vestibular system’s role on cognition, such as perceptual/visuospatial ability, memory, attention, and executive function. Knowing the cognitive function of individuals with vestibular dysfunction facilitates the setting of vestibular rehabilitation therapy goals. Evidence reveals that in patients with intractable dizziness following vestibular rehabilitation there is a significant improvement in vestibular function and cognitive function including attention, visuospatial ability and executive function with coincidental improvement in DHI. ^31^ The findings of this study are circumscribed to oddball auditory tasks only, which might be a limitation. The majority of participants in the present study had mild handicap, this might have an influence on the findings.

## Conclusions

In the present study, the P300 was absent in a greater number of individuals with vestibular dysfunction, suggesting cognitive impairment. However, when the P300 was present, the peak latency and amplitude were not significantly different in both groups.

## Data availability

### Underlying data

Mendeley Data: Underlying data for ‘Cortical auditory potentials and cognitive potentials in individuals with and without vestibular dysfunction’ https://www.doi.org/10.17632/hn6z8x5vkk.1. ^21^

This project contains the following underlying data:

• Data file 1. Description.txt
• Data file 2. Event related potentials in individuals with vestibular dysfunction.xlsx

Data are available under the terms of the Creative Commons Attribution 4.0 International license (CC-BY 4.0).

## Consent

Written informed consent for publication of the participants’ details was obtained from the participants.
