# Supplementary material for: Cortical auditory potentials and cognitive potentials in individuals with and without vestibular dysfunction
Source: F1000Res. 2023 Oct 18;11:1013. Originally published 2022 Sep 7. [Version 3] doi: 10.12688/f1000research.122677.3 (PMC10457561; doi:10.12688/f1000research.122677.3)
Supplement: Peer review history

# Reviewer report: Reviewer response for version 3

**DOI:** 10.5256/f1000research.157492.r216594

**Published:** 2023-11-22

**Authors:** Aravind Kumar Rajasekaran

**Affiliations:** 1 Speech Pathology and Audiology, National Institute of Mental Health and Neurosciences, Bengaluru, Karnataka, India

Authors may consider the following

 1. Authors have already given the basis on which they had differentiated peripheral and central vestibular conditions as a response to my previous query. The same can be incorporated into the manuscript. Similar inclusions can be done if deemed fit. 

 2. A different terminology to mark the negativity post-P3. 

 Beyond that, I have no further comments to make.

Is the work clearly and accurately presented and does it cite the current literature?

Partly

If applicable, is the statistical analysis and its interpretation appropriate?

Yes

Are all the source data underlying the results available to ensure full reproducibility?

Yes

Is the study design appropriate and is the work technically sound?

Yes

Are the conclusions drawn adequately supported by the results?

Partly

Are sufficient details of methods and analysis provided to allow replication by others?

Yes

Reviewer Expertise:

Auditory processing, Electrophysiology, Auditory Neuroscience , Vestibular science

I confirm that I have read this submission and believe that I have an appropriate level of expertise to confirm that it is of an acceptable scientific standard.

# Reviewer report: Reviewer response for version 2

**DOI:** 10.5256/f1000research.140797.r191649

**Published:** 2023-08-25

**Authors:** Aravind Kumar Rajasekaran

**Affiliations:** 1 Speech Pathology and Audiology, National Institute of Mental Health and Neurosciences, Bengaluru, Karnataka, India

**Section: Introduction**

1. **Manuscript:** Different areas of the brain that provide the generation of P300 response include subcortical structures, auditory regions in the cortex and frontal lobe and various association areas neocortex.
**Suggestion:** Rephrase - Different areas of the brain that generate P300 response include subcortical structures, auditory cortical regions, frontal lobe and various association areas.
2. **Manuscript: **The subcomponents for P300, P3a is generated from the frontal working memory which helps in early attention whereas P3b is an attention-driven stimulus generated from the temporal and parietal structures.
**Suggestion: **Rephrase - The subcomponents of P300, P3a and P3b are generated by different neural structures. The frontal lobe is the primary generator of P3a, reflecting the working memory, which helps in early attention. The P3b reflects an attention-driven stimulus-generated response and is generated from the temporal and parietal structures.
3. **Manuscript: **In the case of individuals with vertigo, the literature has reported reduced behavioural cognitive abilities
**Suggestion: **Rephrase - ****Behavioural cognitive abilities are reported to be affected in individuals with vertigo.
4. **Manuscript: **A national health and nutrition examination survey done in the US revealed an association between vestibular and cognitive function in the adult population.5 There is a steady accumulation of evidence that the vestibular lesion leads to a cognitive deficit.
**Suggestion: **May delete redundant information and mark it as a reference in the previous sentence.
5. **Manuscript: **It is not essentially directly related to reflexive signs and perceptual disturbances associated with vestibular dysfunction.
**Suggestion: **Does not fit in the flow of information. Rephrase.
6. **Manuscript: **And there is minimal scientific evidence on cortical potentials in individuals with vestibular dysfunction.
**Suggestion: **Appears to be a sudden statement. Again affecting the flow. Rephrase.
7. **Manuscript: **Hence, the current study aims to compare the findings of cortical potentials (P1, N1, P2, and N2) and cognitive potentials (P300 and N4) between individuals with and without vestibular dysfunction. The objective was to investigate the relationship of cortical and cognitive potentials peak latency and peak amplitude in individuals with and without vestibular dysfunction and the correlation between DHI score with P300 findings among individuals with vestibular dysfunction.
**Suggestion/Query: **The ‘need statement’ should be written as a derivative of the previous statements in the article. Where is the need to have cortical potentials? The author can explicitly mention the reason. Further, N4 does not fit the narrative.

**Section: Method**

1. **Manuscript: **The patients having abnormal findings on oculomotor examination or VEMPs assessment was considered for this study.
**Query: **Why either?
2. **Manuscript: **Twenty individuals with vestibular dysfunction in group I included 12 individuals diagnosed with peripheral vestibular lesion and eight individuals with central vestibular lesion **.**
**Query: **What central conditions?
3. **Manuscript:** Two non-inverting electrodes were placed on the scalp, one on the vertex (Cz) and the other on the parietal lobe (Pz).
**Suggestion:** Rephrase. Two non-inverting electrodes were placed on the scalp, one on the vertex (Cz) and the other on the parietal (Pz).
4. **Manuscript:** And the negativity peak following the P300 was considered as N4 (late negativity)
**Query:** The author may state why they have included N4 in the method. This does not appear to be the aim or the objective of the study. Further, it is not even marked in the waveforms.

**Section: Results**

1. **Manuscript:** The mean amplitude of P300 was larger in group I compared to group II, this finding is controversial
**Suggestion: **Use a different term instead of ‘...controversial...’

**Section:** **Discussion**

1. **Manuscript:** **Para 2: **These findings are not in agreement with results of earlier investigation
**Suggestion:** The authors refer to an article which evaluated patients with Tinnitus, why so?
2. **Manuscript:** Para 3: In contrast to the findings of the present study, earlier investigations have reported prolonged latency for P300 in individuals with vestibular dysfunction compared to the control group. ^20,22^
**Query: **The articles referred to by the author is not related to the vestibular dysfunction.
3. **Manuscript:** The contrasting findings observed in the present study and earlier investigations could be because of differences in the site of vestibular lesion across studies. Studies in the literature have included individuals with peripheral vestibular lesions where the site of the lesion was localized to the lateral semicircular canal.
**Query: **Referred studies have not done VEMP, and the current study did not perform caloric.
4. **Manuscript:** The majority of the participants with peripheral vestibular lesion in the present study were found to have mild handicap based on DHI scores. The degree of handicap might have an influence on the latency and amplitude of P300.
**Query: **How did authors come to this conclusion?

Is the work clearly and accurately presented and does it cite the current literature?

Partly

If applicable, is the statistical analysis and its interpretation appropriate?

Yes

Are all the source data underlying the results available to ensure full reproducibility?

Yes

Is the study design appropriate and is the work technically sound?

Yes

Are the conclusions drawn adequately supported by the results?

Partly

Are sufficient details of methods and analysis provided to allow replication by others?

Yes

Reviewer Expertise:

Auditory processing, Electrophysiology, Auditory Neuroscience , Vestibular science

I confirm that I have read this submission and believe that I have an appropriate level of expertise to confirm that it is of an acceptable scientific standard, however I have significant reservations, as outlined above.

# Response

**Published:** 2023-10-14

**Authors:** Anupriya Ebenezer

**Affiliations:** Manipal Academy of Higher Education, India

We thank you for reviewing the manuscript and giving your valuable comments. We have made the necessary modifications as per the feedback and answered few queries. The modifications are listed below;

**Section: Introduction**

**1. Manuscript:** Different areas of the brain that provide the generation of P300 response include subcortical structures, auditory regions in the cortex and frontal lobe and various association areas neocortex.

**Suggestion:** Rephrase - Different areas of the brain that generate P300 response include subcortical structures, auditory cortical regions, frontal lobe and various association areas.

**Response:** Appropriate changes made.

**2. Manuscript: **The subcomponents for P300, P3a is generated from the frontal working memory which helps in early attention whereas P3b is an attention-driven stimulus generated from the temporal and parietal structures.

**Suggestion: **Rephrase - The subcomponents of P300, P3a and P3b are generated by different neural structures. The frontal lobe is the primary generator of P3a, reflecting the working memory, which helps in early attention. The P3b reflects an attention-driven stimulus-generated response and is generated from the temporal and parietal structures.

**Response:** Appropriate changes made.

**3. Manuscript: **In the case of individuals with vertigo, the literature has reported reduced behavioural cognitive abilities

**Suggestion: **Rephrase - ****Behavioural cognitive abilities are reported to be affected in individuals with vertigo.

**Response:** Appropriate changes made.

**4. Manuscript: **A national health and nutrition examination survey done in the US revealed an association between vestibular and cognitive function in the adult population.5 There is a steady accumulation of evidence that the vestibular lesion leads to a cognitive deficit.

**Suggestion: **May delete redundant information and mark it as a reference in the previous sentence.

**Response:** Appropriate changes made.

**5. Manuscript: **It is not essentially directly related to reflexive signs and perceptual disturbances associated with vestibular dysfunction.

**Suggestion: **Does not fit in the flow of information. Rephrase.

**Response: **Appropriate changes made.

**6. Manuscript: **And there is minimal scientific evidence on cortical potentials in individuals with vestibular dysfunction.

**Suggestion: **Appears to be a sudden statement. Again affecting the flow. Rephrase.

**Response:** Appropriate changes made.

**7. Manuscript: **Hence, the current study aims to compare the findings of cortical potentials (P1, N1, P2, and N2) and cognitive potentials (P300 and N4) between individuals with and without vestibular dysfunction. The objective was to investigate the relationship of cortical and cognitive potentials peak latency and peak amplitude in individuals with and without vestibular dysfunction and the correlation between DHI score with P300 findings among individuals with vestibular dysfunction.

**Suggestion/Query: **The ‘need statement’ should be written as a derivative of the previous statements in the article. Where is the need to have cortical potentials? The author can explicitly mention the reason. Further, N4 does not fit the narrative.

**Response: ** For analysis we considered the ERP waves till 500 msec duration. ****And we thought of showing the data as per the ERPs peaks analyzed so we included N4 which was present in few individuals in both the groups even thought it was not under our objective. And also we have made the appropriate changes required to maintain the flow of information.

**Section: Method**

**1. Manuscript: **The patients having abnormal findings on oculomotor examination or VEMPs assessment was considered for this study.

**Query: **Why either? (You have to answer this..)

**Response: **All the participants had undergone VEMP and VNG testing. Few patients who had normal VEMPs findings showed abnormal oculomotor function tested using VNG and few patients had findings in reverse. Based on their vestibular test reports and the complaints they were categories under peripheral or central vestibular lesion accordingly.

**2. Manuscript: **Twenty individuals with vestibular dysfunction in group I included 12 individuals diagnosed with peripheral vestibular lesion and eight individuals with central vestibular lesion **.**

**Query: **What central conditions?

**Response: **Especially patients diagnosed with central vestibular lesion were referred from Department of Neurology with MRI report indicative of either cerebellar atrophy, vestibular schwannoma or acoustic neuroma and also indicated abnormal vestibular findings. Considering vestibular assessment, the patients were having abnormal sacculo-collic reflex and/or utriculo-ocular reflex with prolonged latencies or absent cVEMP and /or oVEMP reflexes. And also with delayed saccadic movement, abnormal tracking gain and abnormal slow phase velocity in optokinetic test at 20°/sec and 40°/sec.

**3. Manuscript:** Two non-inverting electrodes were placed on the scalp, one on the vertex (Cz) and the other on the parietal lobe (Pz).

**Suggestion:** Rephrase. Two non-inverting electrodes were placed on the scalp, one on the vertex (Cz) and the other on the parietal (Pz).

**Response:** Appropriate changes made.

**4. Manuscript:** And the negativity peak following the P300 was considered as N4 (late negativity)

**Query:** The author may state why they have included N4 in the method. This does not appear to be the aim or the objective of the study. Further, it is not even marked in the waveforms.

**Response: **The negative peak following the P300 is generally referred as late negativity, we have labelled it as N4. Comparison of late negativity was not an objective of the study. We have labelled the peak N4 in the averaged waveforms.

**Section: Results**

**1. Manuscript:** The mean amplitude of P300 was larger in group I compared to group II, this finding is controversial

**Suggestion: **Use a different term instead of ‘...controversial...’

**Response:**  Appropriate changes made.

**Section: Discussion**

**1. Manuscript:** **Para 2: **These findings are not in agreement with results of earlier investigation

**Suggestion:** The authors refer to an article which evaluated patients with Tinnitus, why so?

**Response: **It was a referencing error by mistake. We have corrected it.

**2. Manuscript:** Para 3: In contrast to the findings of the present study, earlier investigations have reported prolonged latency for P300 in individuals with vestibular dysfunction compared to the control group. ^20,22^

**Query: **The articles referred to by the author is not related to the vestibular dysfunction.

**Response:**  There was in text citation numbering error while modifying the version 1 manuscript, we are sorry for that. Correction done.

**3. Manuscript:** The contrasting findings observed in the present study and earlier investigations could be because of differences in the site of vestibular lesion across studies. Studies in the literature have included individuals with peripheral vestibular lesions where the site of the lesion was localized to the lateral semicircular canal.

**Query: **Referred studies have not done VEMP, and the current study did not perform caloric.

**Response:**  There are no studies available specifically with abnormal VEMP findings. Majority of the studies was done on individuals with abnormal caloric findings and /or oculomotor findings. Caloric test mainly assesses the lateral semicircular canal whereas, VEMP assess the otolith function (utricle and saccule). Though both the lateral semicircular canal and the otolith organ belong to the peripheral vestibular system but their functionalities are different.

**4.Manuscript:** The majority of the participants with peripheral vestibular lesion in the present study were found to have mild handicap based on DHI scores. The degree of handicap might have an influence on the latency and amplitude of P300.

**Query: **How did authors come to this conclusion?

**Response: **A recent study by Ma et al, 2023 have found a correlation between the ERPs and different degrees of DHI severity. Indicating a direct proportionality that higher the degree of dizziness, the higher the cognitive impairment.

# Reviewer report: Reviewer response for version 2

**DOI:** 10.5256/f1000research.140797.r155890

**Published:** 2022-12-05

**Authors:** Prawin Kumar

**Affiliations:** 1 University of Mysore, Mysore, Karnataka, India; 2 Department of Audiology, All India Institute of Speech and Hearing, Mysore, Karnataka, India

The answer to the comments/queries raised is satisfactory. There are no additional comments. The revised manuscript is acceptable from my side.

Is the work clearly and accurately presented and does it cite the current literature?

Partly

If applicable, is the statistical analysis and its interpretation appropriate?

Yes

Are all the source data underlying the results available to ensure full reproducibility?

Yes

Is the study design appropriate and is the work technically sound?

Yes

Are the conclusions drawn adequately supported by the results?

Yes

Are sufficient details of methods and analysis provided to allow replication by others?

Yes

Reviewer Expertise:

Diagnostic Audiology including Electrophysiology

I confirm that I have read this submission and believe that I have an appropriate level of expertise to confirm that it is of an acceptable scientific standard.

# Reviewer report: Reviewer response for version 1

**DOI:** 10.5256/f1000research.134699.r149808

**Published:** 2022-10-18

**Authors:** Prawin Kumar

**Affiliations:** 1 University of Mysore, Mysore, Karnataka, India; 2 Department of Audiology, All India Institute of Speech and Hearing, Mysore, Karnataka, India

Authors formulated the research very well except for a few observations.

1. In Abstract, authors can add N4 potential. 
2. In last paragraph of Introduction section, it should be mentioned as Cognitive potentials (P300 and N4) instead of cortical potential.
3. In Method, the rationale for considering only two electrode sites i.e. Cz and Pz should be mentioned.
4. The two groups are considered based on abnormal finding of the VNG & VEMP. No gold standard tests were used to differentiate the peripheral and central lesions. That could also be one of the reasons for minimal differences in the performance between two groups for ALLR and P300. 
5. Overall, preliminary finding of the present study throws light for researchers to explore more comprehensive behavioral cognitive assessment along with electrophysiological measures such as multi-channel ERP recording. 

Is the work clearly and accurately presented and does it cite the current literature?

Partly

If applicable, is the statistical analysis and its interpretation appropriate?

Yes

Are all the source data underlying the results available to ensure full reproducibility?

Yes

Is the study design appropriate and is the work technically sound?

Yes

Are the conclusions drawn adequately supported by the results?

Yes

Are sufficient details of methods and analysis provided to allow replication by others?

Yes

Reviewer Expertise:

Diagnostic Audiology including Electrophysiology

I confirm that I have read this submission and believe that I have an appropriate level of expertise to confirm that it is of an acceptable scientific standard.

# Response

**Published:** 2022-11-05

**Authors:** Anupriya Ebenezer

**Affiliations:** Manipal Academy of Higher Education, India

1. Appropriate changes incorporated.
2. Appropriate changes incorporated.
3. The clinical AEP instrument which we used for the study has the facility to perform only 2 channel recording. So, EEG has been recorded for 2 electrode sites. Literature studies have shown greater amplitude for P300 at Cz and Pz electrode sites. Therefore, Cz and Pz electrode sites were selected to record P300.
4. Group I was selected based on the vestibular test findings. Group II was normal healthy individuals with no complaint of vertigo. The vestibular assessment test battery included subjective vestibular assessment, VEMP, and VNG.
5. Thank you for the encouraging words.

# Reviewer report: Reviewer response for version 1

**DOI:** 10.5256/f1000research.134699.r149811

**Published:** 2022-09-13

**Authors:** Sangamanatha Ankmnal Veeranna

**Affiliations:** 1 College of Nursing and Health Professions, School of Speech and Hearing Sciences, J.B. George Building, University of Southern Mississippi, Hattiesburg, MS, USA

**Abstract:**

 The whole abstract should be rewritten so that the readers can follow. It is not clear whether P300 should be used to understand cognitive deficits in individuals with vestibular dysfunction.

 The Results section should be rewritten:

- In the manuscript, the authors have mentioned that there are no statistical differences in the amplitude between Cz and Pz but in the abstract authors have mentioned that there is a significant difference.
- If there are no statistically significant differences in the P300 amplitude, why are the authors discussing this?  

**Introduction:**

- I would not use “ *etc*.” in a manuscript.
- There are sections where authors should provide references. For example, “ *Further, the majority of individuals with vestibular dysfunction are also found to have anxiety and depression.*”

**Methods:**

 A lot of key information is missing in the Methods section.

- For Group I, did the authors assess oculomotor function and VEMP or both?
- The age range of Group I and Group II? How many male and female participants were included?
- Authors have vaguely mentioned that participants in Group 2 had peripheral and central vestibular lesions. Authors should provide more details on peripheral vs central lesions.
- The authors reported that all participants' hearing thresholds were within normal limits. Do the authors mean < 15 dB HL or 20 dB HL? Some participants were 60 years old, there could be some age-related hearing loss in these participants. The authors should provide hearing thresholds for all the participants (control and experimental groups).
- What was the artifact rejection?
- The authors stated that “ *The participants were instructed to count the target stimuli and report at the end of the recording*”, did participants correctly count the target stimuli? The authors should add this information.
- Was all the testing carried out in one session or multiple sessions?

**Data analysis:**

- Who marked the peaks?
- The authors carried out the statistical analysis using multiple t-tests. But the authors did not include age and hearing thresholds in their analysis. The authors should use different statistical analyses.

**Discussion:**

 I was not happy with the discussion section; I feel it is all over the place.

- Paragraph 1: Most of this content should be in the introduction not in the discussion.
- Paragraph 2: The authors did not report hearing thresholds, or they did not include hearing thresholds in the statistical analysis.
- Paragraph 3: The authors reported that 30-40% of individuals with vestibular dysfunction demonstrated an absence of P300, is this statistically significant? The Chi-square test may help in determining whether it is statistically significant or not.
- The authors are talking about cVEMP and oVEMP in the discussion, but they did not talk about these measures in the method.
- The authors are also discussing the severity of dizziness, which was not mentioned in the Methods section.

**Limitations:**

 Authors should list limitations.

**Conclusions:**

 The authors should write the conclusion based on findings from this study. I do not see that conclusion here. This section should be rewritten.

Is the work clearly and accurately presented and does it cite the current literature?

Yes

If applicable, is the statistical analysis and its interpretation appropriate?

Partly

Are all the source data underlying the results available to ensure full reproducibility?

Yes

Is the study design appropriate and is the work technically sound?

Partly

Are the conclusions drawn adequately supported by the results?

No

Are sufficient details of methods and analysis provided to allow replication by others?

No

Reviewer Expertise:

Auditory Evoked Potentials, Auditory Processing Disorders, Psychoacoustics

I confirm that I have read this submission and believe that I have an appropriate level of expertise to confirm that it is of an acceptable scientific standard, however I have significant reservations, as outlined above.

# Response

**Published:** 2022-11-05

**Authors:** Anupriya Ebenezer

**Affiliations:** Manipal Academy of Higher Education, India

**Abstract:**

 Thank you for the suggestions. As per the suggestion, the abstract was rewritten.

- *In the manuscript, the authors have mentioned that there are no statistical differences in the amplitude between Cz and Pz but in the abstract authors have mentioned that there is a significant difference.*

Appropriate changes were made.

- *If there are no statistically significant differences in the P300 amplitude, why are the authors discussing this?  *

The abstract was rewritten with appropriate changes.

**Introduction:**

- *I would not use “etc.” in a manuscript.*

Appropriate changes incorporated.

- *There are sections where authors should provide references. For example, “Further, the majority of individuals with vestibular dysfunction are also found to have anxiety and depression.”*

Respective citation added, and appropriate changes were made in the Reference section.

**Methods:**

*A lot of key information is missing in the Methods section.*

- *For Group I, did the authors assess oculomotor function and VEMP or both?*

Yes, both the oculomotor function test (VNG) and VEMP (cVEMP and oVEMP) were performed to rule out vestibular dysfunction in Group I.

- *The age range of Group I and Group II? How many male and female participants were included?*

Details are added in the Method section first paragraph.

- *Authors have vaguely mentioned that participants in Group 2 had peripheral and central vestibular lesions. Authors should provide more details on peripheral vs central lesions.*

Group II included individuals with no vestibular dysfunction.

- *The authors reported that all participants' hearing thresholds were within normal limits. Do the authors mean < 15 dB HL or 20 dB HL? Some participants were 60 years old, there could be some age-related hearing loss in these participants. The authors should provide hearing thresholds for all the participants (control and experimental groups).*

The mean and standard deviation of the hearing threshold of the participants are added in the Method section under the heading 'Participants'.

- *What was the artifact rejection?*

Sweeps with amplitude greater than ±50µV were rejected from averaging. The same has been added to the manuscript.

- *The authors stated that “The participants were instructed to count the target stimuli and report at the end of the recording”, did participants correctly count the target stimuli? The authors should add this information.*

Yes, all subjects had a practice trial on the task before ERPs were recorded. The same has been added to the manuscript under the Method section.

- *Was all the testing carried out in one session or multiple sessions? *

Yes, all the testing was carried out in one session.

**Data analysis:**

- *Who marked the peaks?*

Peaks were identified separately by 3 investigators. Peaks were considered to be present when the markings of 2 investigators were in agreement

- *The authors carried out the statistical analysis using multiple t-tests. But the authors did not include age and hearing thresholds in their analysis. The authors should use different statistical analyses.*

Descriptive statistical analysis (mean and SD) was done for both age and hearing thresholds. Descriptive statistics of age, as well as the hearing threshold, have been added to the Method section.

**Discussion:**

*I was not happy with the discussion section; I feel it is all over the place.*

 Thank you for the suggestion. The Discussion section is modified to improve the flow of information.

- *Paragraph 1: Most of this content should be in the introduction not in the discussion.*

Appropriate changes incorporated.

- *Paragraph 2: The authors did not report hearing thresholds, or they did not include hearing thresholds in the statistical analysis.*

Statistical analysis of the hearing threshold is added to the Method section first paragraph.

- *Paragraph 3: The authors reported that 30-40% of individuals with vestibular dysfunction demonstrated an absence of P300, is this statistically significant? The Chi-square test may help in determining whether it is statistically significant or not.*

Thank you for the suggestion. We performed a chi-square test and a significant difference was observed (X ^2^(1) = 8.53, p= 0.003) with the odd ratio 15.83. The same has been added to the manuscript under the Result section.

- *The authors are talking about cVEMP and oVEMP in the discussion, but they did not talk about these measures in the method.*

cVEMP and oVEMP were administered for the Group I participant selection. The same is mentioned in the Method.

- *The authors are also discussing the severity of dizziness, which was not mentioned in the Methods section.*

By severity, we were speaking about the dizziness handicap. The word 'severity' is replaced with the word 'handicap'. Appropriate changes were incorporated for better understanding.

**Limitations:**

*Authors should list limitations.*

 Some of the limitations were added to the Discussion in the last paragraph.

**Conclusions:**

*The authors should write the conclusion based on findings from this study. I do not see that conclusion here. This section should be rewritten.*

 The conclusion is rewritten with appropriate changes.
